# The role of gut microbiota in myocardial ischemia-reperfusion injury

**DOI:** 10.3389/fcvm.2025.1625299

**Published:** 2025-09-23

**Authors:** Xin Chen, Lu Ye, Xin Zou, Yuan Zhou, Chan Peng, Rui Huang

**Affiliations:** ^1^ Cardiovascular Disease Center, The Central Hospital of Enshi Tujia and Miao Autonomous Prefecture, Enshi, Hubei, China; ^2^ Hubei Selenium and Human Health Institute, The Central Hospital of Enshi Tujia and Miao Autonomous Prefecture, Enshi, Hubei, China; ^3^ Hubei Provincial Key Lab of Selenium Resources and Bio Applications, Enshi, Hubei, China; ^4^ Cardiovascular Disease Center, The Central Hospital of Enshi Tujia and Miao Autonomous Prefecture, Hubei Minzu University, Enshi, Hubei, China; ^5^ Department of Nephrology and Endocrinology, The Lichuan Ethnic Hospital of Traditional Chinese Medicine, Lichuan, Hubei, China; ^6^ Department of Nephrology and Endocrinology, The People’s Hospital of Lichuan City, Lichuan, Hubei, China; ^7^ Department of Pediatrics, The Third People’s Hospital of Yichang, Yichang, Hubei, China

**Keywords:** gut microbiota, myocardial ischemia, reperfusion injury, inflammation, gut metabolites

## Abstract

Myocardial ischemia-reperfusion injury denotes the pathological damage resulting from the restoration of blood flow and oxygen supply following acute coronary artery occlusion. Myocardial ischemia-reperfusion injury is commonly seen in acute coronary syndromes and is an important factor in the development of ischemic cardiomyopathy, which severely affects the prognosis of coronary heart disease. The gut microbiota, a complex ecosystem with multifaceted functions, plays a crucial role in host health. Dysregulation of the gut microbiota exerts substantial effects on the onset and progression of cardiovascular diseases, including myocardial ischemia-reperfusion injury. This review elucidates the mechanisms underlying myocardial ischemia-reperfusion injury and the involvement of the gut microbiota in this process, encompassing aspects such as intestinal barrier integrity, microbial dysbiosis, inflammatory responses, oxidative stress, mitochondrial dysfunction, and metabolic alterations. Additionally, we investigate various interventions that modulate myocardial ischemia-reperfusion injury by influencing the gut microbiota. Maintaining a healthy intestinal barrier and a stable microbial ecology is paramount in preventing myocardial ischemia-reperfusion injury. High-fiber diets, probiotic consumption, short-chain fatty acids supplementation, and Traditional Chinese Medicine, can safeguard the heart against myocardial ischemia-reperfusion injury by regulating gut microbiota through diverse mechanisms. As the role of gut microbiota in myocardial ischemia-reperfusion injury continues to be investigated, it provides important therapeutic targets and drug development opportunities for the prevention and treatment of myocardial ischemia-reperfusion injury. However, further in-depth and comprehensive studies are required to fully realize these potentials.

## Introduction

Myocardial ischemia/reperfusion (I/R) typically occurs in myocardial ischemia induced by acute coronary artery occlusion. Ischemic myocardium can be salvaged by reperfusion of the coronary arteries via percutaneous coronary intervention (PCI), coronary artery bypass grafting (CABG) or thrombolytic therapy. Nevertheless, myocardial reperfusion may subsequently exacerbate and accelerate myocardial injury, known as myocardial ischemia-reperfusion injury (MIRI) (1). MIRI can mediate adverse remodeling of the myocardium, cardiac dysfunction, and arrhythmia, which can progress to ischemic heart disease, further deteriorating the heart and influencing the prognosis and lifespan of the patient (2).

The severity of MIRI is dependent on the duration of ischemia, the extent of the ischemic area, the reperfusion blood flow, oxygen content, and other risk factors (3). The mechanism of MIRI is complex and diverse, involving various biological processes such as different types of cell death, autophagy, inflammation, oxidative stress, mitochondrial damage, energy metabolism disorders, and ion homeostasis disorders (4, 5). The research on the mechanism and treatment of MIRI is increasing annually. Recent studies have discovered that the gut microbiome is closely associated with MIRI (6, 7), but the mechanisms and interactions have not been fully clarified.

The gut microbiota represents the most abundant symbiotic microbial community within the human body, exerting significant influence on both human health and the progression of various diseases. It plays a critical and multifaceted role in immune regulation and metabolic homeostasis, contributing to essential physiological processes such as gene expression, substance metabolism, anti-inflammation, gastrointestinal hormone regulation, and mental health maintenance (8–10). The physical and immune barriers of the intestine can be altered by the gut microbiota, creating opportunities for interaction between the intestine and other systems and organs. Concurrently, the gut microbiota is capable of producing a diverse array of metabolic products that play essential regulatory roles in maintaining the host's health and physiological homeostasis (11, 12). Substantial evidence currently demonstrates that the gut microbiota plays an important role in diseases such as cardiovascular disease, renal disease, neurological disease, diabetes, obesity, and sepsis (13–17).

The composition and biological activity of the gut microbiota are influenced by genetic and environmental factors, such as infection, diet, stress, and antibiotic use, leading to gut microbiota dysbiosis (18, 19). Gut microbiota dysbiosis is closely linked to a wide range of gastrointestinal, metabolic, neurological, and inflammatory disordersp. The gut-heart axis represents an emerging area in cardiovascular research. This bidirectional communication system underscores the complex and dynamic interactions between the gastrointestinal tract and the cardiovascular system, which are mediated through multiple signaling pathways involving microbial metabolites, immune modulation, inflammatory processes, and neurohumoral mechanisms (13, 20). Recent studies have implicated the gut microbiota in the pathogenesis and prognosis of heart failure, myocardial fibrosis, myocardial infarction, and arrhythmia (21–24). However, the precise role of gut microbiota in MIRI remains incompletely understood. This review aims to elucidate the intricate relationship between gut microbiota and MIRI.

## Mechanisms of myocardial ischemia-reperfusion injury

MIRI is an inevitable pathological process characterized by a complex underlying mechanism. Multiple forms of cell death are among the primary drivers of MIRI (Table 1). Early studies have demonstrated that apoptosis is the first form of cell death observed in MIRI. Upon myocardial reperfusion, the sudden reintroduction of oxygen leads to a substantial increase in intracellular reactive oxygen species (ROS), which triggers cardiomyocyte apoptosis (4). Subsequent research has identified additional modes of cell death involved in MIRI, including ferroptosis, pyroptosis, and necroptosis (41–43). Under conditions of coronary artery ischemia and reperfusion, iron levels rise sharply, contributing to myocardial dysfunction, which is primarily attributed to the excessive generation of free radicals (44). Lipid peroxidation serves as a hallmark of ferroptosis, while iron overload functions as a significant inducer of this process (41). Pyroptosis is an inflammatory form of cell death (42), whereas necroptosis is characterized by the phosphorylation of receptor-interacting serine/threonine-protein kinase 3 (43). These cell deaths adversely affect both short-term and long-term cardiac remodeling function after myocardial I/R (45).

**Table 1:** Interventions affecting MIRI through modulation of gut microbiota function.

Treatments	Variety	Alterations in gut microbiota composition	Alterations in gut microbiota metabolites	Proof of concept	References
Diet	Mediterranean diet, High-fiber diet, C3G-rich diet	↑Bacteroides acidifaciens, ↑Bifidobacterium	↑Short chain fatty acid acetate, ↓TMAO	Improving mitochondrial function, Anti-inflammatory	Gantenbein et al. (25); Marques et al. (26); Kaye et al. (27); Trinei et al. (28)
Metabolic regulation	Phenylacetylglycine, Anti-TMAO substance, Urolithin B	↓F/B, ↑Anaerobes	↑PAGly, ↓TMAO, ↑Urolithin B	Improving mitochondrial function, Inhibiting ferroptosis, Antiapoptosis, Antioxidant stress	Wang et al. (6); Xu et al. (29); Videja et al. (93); Zheng et al. (30)
Synbiotic	Prebiotics, Probiotic	↑Lactobacillus. reuteri, ↑Bifidobacterium infantis, ↓Proteobacteria, ↑Bacteroidetes, ↑Actinobacteria,	↑GABA, ↑Inosine,↓LPS	Antiapoptosis, Anti-inflammatory	Oniszczuk et al. (31); Wang et al. (32); Zhang et al. (33); Borshchev et al. (34); Bulut et al. (35)
Short chain fatty acids	Acetate, Propionate, Butyrate	↑Clostridium_*sensu*_*stricto*_1, ↑Cetobacterium, ↑Lactococcus	↓Triglyceride, ↑Carbohydrate, ↑Cofactor, ↑Vitamin, ↑Amino acid	Sympathetic inhibition, antioxidant stress, Anti-inflammatory	Liu et al. (36); Yu et al. (37); Deng et al. (38)
Traditional Chinese Medicine	FMN, Simiao Yongan Decoction, Electroacupuncture	↑Ligilactobacillus, ↑Coprococcus, ↑Blautia, ↑Muribaculaceae, ↓F/B, ↓Spirochaetota, ↓Campylobacterota	↓TNF-α, ↓NF-*κ*B, ↓LPS	Repairing the gut barrier, Anti-inflammatory	Zhang et al. (39); Cui et al. (40); Bai et al. (7)

Another crucial factor implicated in MIRI is the inflammatory response (1). Prior research has demonstrated that although inflammation is triggered during myocardial ischemia, the restoration of blood flow and oxygenation results in the generation of substantial ROS by cardiomyocytes and the release of key inflammatory mediators, such as interleukins, neutrophils, and inflammasomes, which are pivotal in initiating and sustaining the inflammatory phase of MIRI (46–48). Additionally, macrophages and circulating leukocytes also contribute to this inflammatory response (49). The NLR family pyrin domain containing 3 (NLRP3) inflammasome serves as a critical link between chronic inflammation and the inflammatory process in MIRI (50).

Mitochondrial dysfunction and oxidative stress are intricately linked to the pathogenesis of MIRI. Normal mitochondrial function is essential for maintaining cellular homeostasis and ensuring cell survival (51). During myocardial ischemia, the deficiency of oxygen and nutrients leads to the accumulation of lactate and ROS. Upon reperfusion, mitochondria produce excessive ROS, which results in intracellular calcium overload and the activation of apoptotic protein activities, ultimately causing mitochondrial swelling and apoptosis. Excessive ROS can also induce structural damage to cellular proteins, lipids, and deoxyribonucleic acid (DNA), leading to a loss of cellular function and cell death (52–54). Furthermore, ROS can activate pro-inflammatory signaling pathways, triggering the release of cytokines, chemokines, and adhesion molecules, thereby exacerbating inflammation. Damaged cells may also secrete pro-inflammatory factors such as Tumor necrosis factor-α (TNF-α) and Lnterleukin-1β (IL-1β), further increasing ROS production (55). Meanwhile, maintaining an optimal cellular autophagy state is essential for preserving cardiac homeostasis. During the myocardial reperfusion phase, excessive autophagic activity may lead to the degradation of normal organelles and mitochondria, resulting in myocardial cell injury and potentially cell death (56). These complex interactions culminate in sustained and irreversible tissue damage.

## Gut microbiota and myocardial ischemia-reperfusion

### Intestinal barrier and gut microbiota ecology

The human gut microbiota constitutes a complex ecosystem. As research into this area deepens, the physiological functions and roles of the gut microbiota within the body have been progressively elucidated (57). An increasing number of studies have highlighted the significant role of the gut microbiota in cardiovascular diseases (58). The Disruption of the intestinal barrier can lead to dysbiosis of the microbiota, and at the same time, metabolites are released into the bloodstream, activating inflammatory responses. Disruption of gut microbiota can contribute to atherosclerosis, and participate in the pathogenesis of coronary heart disease, myocardial infarction and heart failure (22, 59). Additionally, it has been shown to have a detrimental impact on myocardial fibrosis (21).

The disruption of the intestinal barrier can lead to dysbiosis of the microbiota, and at the same time, metabolites are released into the bloodstream, activating inflammatory responses.

These impacts are mediated through multiple mechanisms, such as the inflammatory response triggered by compromised intestinal barrier function, metabolites produced by the gut microbiota, and the dysbiosis induced by exogenous antibiotics (60). Liu Q et al. (61) demonstrated that irisin can mitigate MIRI by alleviating intestinal dysbiosis, endothelial dysfunction, and exerting anti-inflammatory effects. Some studies have found that gossypin treatment in isoproterenol (ISO)—induced rat MIRI model can prevent the disruption of the gut microbiota and alter its richness and diversity to protect against MIRI (62). In summary, the disruption of the intestinal barrier and the dysregulation of the gut microbial ecosystem can initiate inflammatory responses and lead to the production of harmful metabolites, which may enter the systemic circulation and contribute to the exacerbation of MIRI.

### Inflammation

Under normal conditions, the intestinal epithelium and immune cells function as a protective barrier for the gastrointestinal tract (63). The gut microbiota plays a crucial role in host immune regulation, with its metabolites modulating the activity of immune cells and the production of proinflammatory cytokines (64). However, under conditions such as inflammation, stress, and aging, the intestinal barrier may become more permeable and functionally impaired, leading to microbial translocation and the release of metabolic toxins into the systemic circulation (65). Following myocardial I/R, gut microbiota and harmful metabolites can translocate into the bloodstream, stimulating the recruitment of neutrophils, which can directly impact cardiomyocytes and induce apoptosis (66).

Previous studies have demonstrated that lipopolysaccharide (LPS) derived from gram-negative bacilli can enter the systemic circulation when the intestinal barrier is disrupted. Once in circulation, LPS triggers the activation of pathogen-associated molecular patterns (PAMPs), leading to the expression and secretion of cellular inflammatory mediators, which can result in myocardial damage (67). TNF-α has been identified as a key initiator of cardiomyocyte apoptosis and is known to upregulate the expression of endothelial cell adhesion molecules, including intercellular adhesion molecule-1 (ICAM-1) and vascular cell adhesion molecule-1 (VCAM-1). Inhibiting VCAM-1 and ICAM-1 has been shown to reduce neutrophil infiltration and mitigate LPS-induced cardiac injury (68). After the invasion of the gut microbiota by pathogenic bacteria, the integrity of the intestinal barrier is compromised, leading to the release of pro-inflammatory cytokines such as TNF-α and IL-1β, ultimately contributing to myocardial injury (69). Zhang Y et al. (39) reported that a flavin mononucleotide (FMN) with anti-inflammatory properties could suppress inflammation and reduce no-reflow phenomena following myocardial I/R by enhancing the abundance of anti-inflammatory bacteria within the gut microbiota. Dysbiosis of the gut microbiota and the subsequent secretion of harmful metabolites can directly promote the production of neutrophils and activate inflammatory cytokines, leading to myocardial damage and exacerbating MIRI. During the occurrence of MIRI, enhancing the anti-inflammatory capacity of the gut microbiota may help suppress the inflammatory response and alleviate the extent of MIRI.

### Mitochondrial dysfunction and oxidative stress

Oxidative stress, disruption of mitochondrial dynamics, and dysregulation of calcium (Ca^2+^) handling are the key factors contributing to MIRI. Studies have demonstrated that an imbalance in gut Microbiota can result in mitochondrial dysfunction and the activation of oxidative stress. The interplay between gut Microbiota and the host's intestinal epithelial surface can trigger signaling pathways associated with oxidative stress and inflammatory responses (70). Additionally, intestinal pathogens are capable of inducing mitochondrial dysfunction and autophagy in myocardial cells, which may contribute to myocardial dysfunction (71). Recent research has indicated that certain mitochondria-targeted drugs can mitigate MIRI by decreasing succinate accumulation during ischemia, inhibiting succinate oxidation upon reperfusion, reducing ROS mage, preserving Ca^2+^ homeostasis, and modulating mitochondrial dynamics and quality control (58, 72). Cheng G et al. revealed that Gossypin exerts protective effects against cardiac injury induced by MIRI through the modulation of oxidative stress, inflammation, and gut microbiota (62).

### Trimethylamine N-oxide (TMAO)

The gut microbiota plays a crucial role in various host metabolic processes and generates diverse metabolites. The gut microbiota catabolizes choline to produce TMAO, a metabolite identified as a risk factor for metabolic, cardiovascular, and cerebrovascular diseases (73, 74). An imbalance in the gut microbiota can result in elevated synthesis of TMAO, which can alter cholesterol and bile acid metabolism and activate inflammatory pathways (75). Elevated TMAO levels have been associated with the progression of atherosclerosis and an increased incidence of adverse cardiovascular events (76). Reducing TMAO levels can mitigate inflammation by decreasing the level of interleukin-8 (IL-8) and prevent heart failure following myocardial infarction (77). Wang L et al. (6) demonstrated that dapagliflozin reduces TMAO levels, a metabolite generated by the gut microbiota, thereby alleviating ferroptosis in cardiomyocytes post-I/R. Gut metabolites, like TMAO, once released into the bloodstream, can trigger inflammation. During the process of I/R, they can exacerbate atherosclerosis and lead to ferroptosis of cardiomyocytes.

### Short chain fatty acids

Short-chain fatty acids (SCFAs) are metabolites generated by the gut microbiota through nutrient metabolism, which exert significant effects on the host's immune system and overall health. The primary SCFAs include acetate, propionate, and butyrate (78). Acetate has been shown to downregulate the expression of early growth response protein 1 in both the heart and kidney, thereby mitigating inflammation and reducing cardiac and renal fibrosis (26). Additionally, acetate can modulate sympathetic nerve activity, leading to a reduction in blood pressure and heart rate, thus providing cardioprotective benefits (79). The gut microbiota inhibit the histone deacetylase (HDAC) mediated by butyrate by synthesizing SCFAs, thereby reducing the transcriptional activity of the NF-*κ*B pathway and decreasing the inflammatory response. Butyrate can also increase cardiac contractility and reduce arterial tension to enhance cardiac output, enhance the activity of superoxide dismutase-1 in the heart, improve cardiac function and prevent myocardial fibrosis (80, 81). A review by Wang×et al. elucidates the crucial role of SCFAs in mitigating MIRI through their anti-inflammatory and metabolic regulatory functions (82).

## Treatments

**Figure 1:**
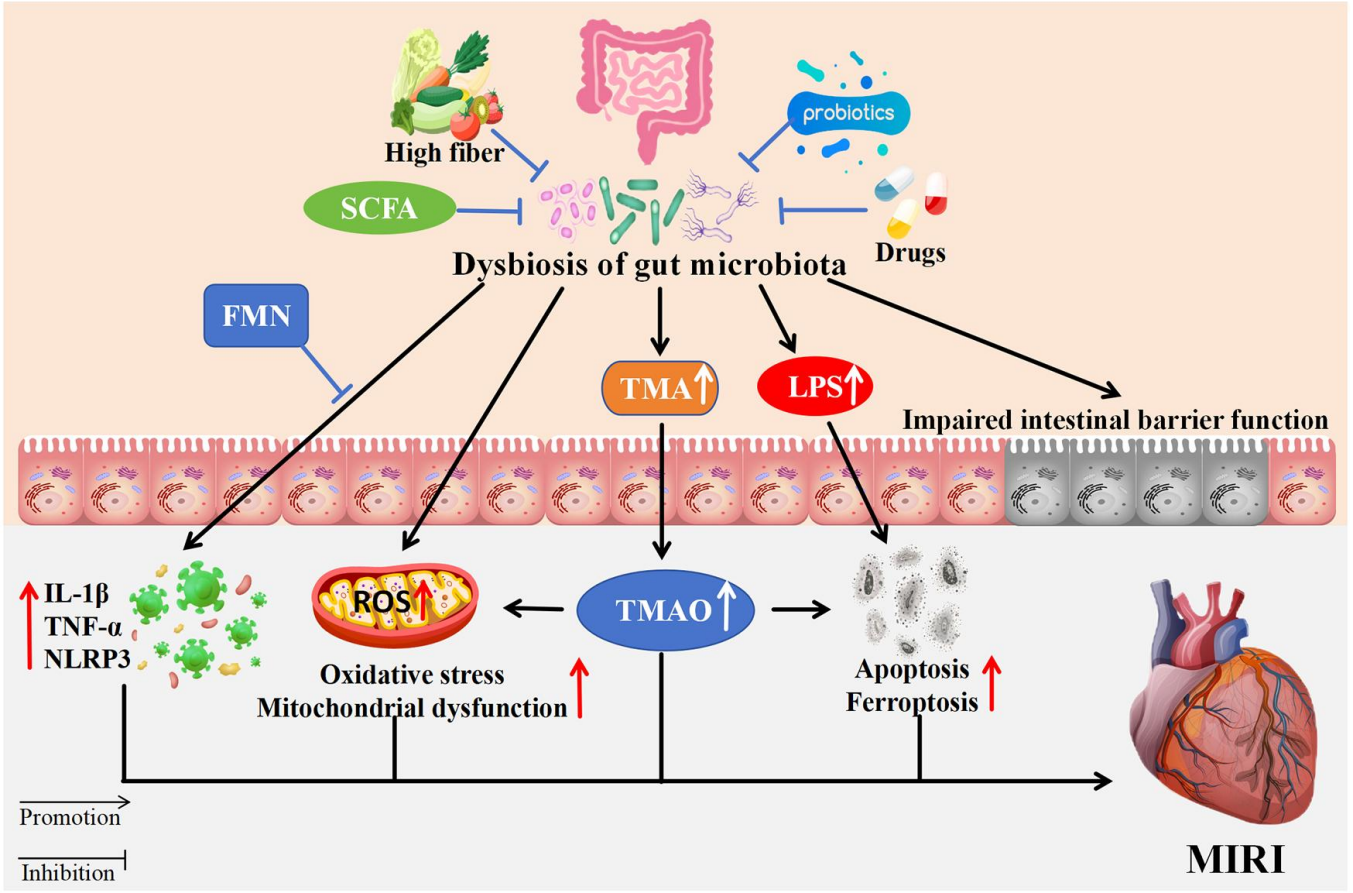
The impact of gut microbiota dysbiosis on MIRI. Dysbiosis contributes to increased TMA and LPS, impairing intestinal barriers. This results in elevated TMAO, oxidative stress, mitochondrial dysfunction, apoptosis, and ferroptosis ultimately affecting MIRI. High fiber, SCFA, probiotics, FMN, and TCM mitigate dysbiosis effects. Arrows indicate promotion or inhibition pathways. SCFA, Short-chain fatty acids; FMN, flavin mononucleotide; TMA, trimethylamine; LPS, lipopolysaccharide; IL-1β, lnterleukin-1β; TNF-α, tumor necrosis factor-α; NLRP3, NLR family pyrin domain containing 3; ROS, reactive oxygen species; TMAO, trimethylamine N-oxide.

Modifying the function of gut microbiota has emerged as a significant approach in the prevention and treatment of various diseases. Various modalities have been found to play a protective role in cardiovascular disease by influencing the gut microbiota, including diet, antibiotics, probiotics, fatty acids, traditional Chinese medicine (TCM), and others (Figure 1).

### Intact gut barrier and balanced microbiota ecosystem

Preserving the integrity of the intestinal barrier and mitigating the ecological disruption of the gut microbiota are crucial for preventing organ and tissue damage resulting from microbial dysbiosis (83). It has been shown that the invasion of pathogenic bacteria can compromise intestinal barrier function, trigger an inflammatory response, and promote the secretion of inflammatory mediators and toxic metabolites, thereby damaging cardiac tissue (58). Intestinal disorders and the inappropriate use of antibiotics can contribute to intestinal barrier dysfunction and microbial dysbiosis (84). Studies have indicated that early antibiotic exposure in infants can disrupt the composition and development of the gut microbiota, potentially influencing body weight and cardiovascular risk throughout life (85). Previous studies have shown that Irisin can alleviate MIRI by reducing gut microbiota imbalance, endothelial dysfunction and anti-inflammatory effects (61). Preserving the integrity of the intestinal barrier and maintaining the ecological balance of the gut microbiota have a significant preventive effect on MIRI.

### Metabolic regulation treatment

Metabolites derived from gut microbiota exert significant influence across various systems and diseases. The gut microbiota metabolite phenylacetylglycine was found to inhibit MIRI-induced cardiomyocyte apoptosis and reduce myocardial infarct size, suggesting a novel therapeutic approach for myocardial infarction patients (29). The compound dapagliflozin has been shown to lower TMAO levels, a metabolite generated by intestinal microorganisms, and consequently modulate associated target genes to alleviate ferroptosis in cardiomyocytes (6). Urolithin B is one of the intestinal metabolites with antioxidant capacity. Urolithin B was found to protect against MIRI by inhibiting autophagy and oxidative stress, reducing the size of myocardial infarction, and attenuating cardiac dysfunction in cardiomyocytes via the P62/keap1/NRF2 signaling pathway (30). Indole-3-acetic acid (IAA), which is derived from intestinal microbiota, can protect cardiomyocytes against ferroptosis following myocardial I/R (86). Du et al. (87) have conducted research which has determined that flavobacterium and its metabolite DAT possess a variety of cardioprotective properties. These have been shown to be effective in counteracting MIRI and have the potential to serve as a preventative treatment option for alleviating MIRI. In summary, recent research has demonstrated that certain metabolites of the gut microbiota can contribute to the mitigation of MIRI, either as a prophylactic measure or as a therapeutic intervention.

### Dietary treatment

Diet can affect human health by altering the gut microbiota. Adherence to the Mediterranean diet has been shown to potentially mitigate the incidence of metabolic syndrome and cardiovascular diseases through its anti-inflammatory and antioxidant properties (25). Consumption of a high-fiber diet leads to an increase in the gut microbiota, which plays a protective role in the development of cardiovascular disease by increasing the production of short-chain fatty acid acetates (88). Research indicates that both high-fiber diets and acetate supplementation can alter the gut microbiota in hypertensive mice, thereby preventing the progression of hypertension and heart failure. Conversely, a low-fiber diet may elevate the risk of hypertension due to a deficiency in SCFAs (26, 27). Trinei M et al. (28). demonstrated that intermittent consumption of a diet rich in cyanidin-3-glucoside can exert a protective effect against MIRI by modulating the gut microbiome.

### Probiotic treatment

Probiotics are capable of maintaining the ecological balance of the gut microbiota, enhancing metabolic processes, modulating immune responses, and contributing to overall human health. They hold a significant position in the prevention and management of cardiovascular diseases (31). Lactobacillus can protect the MIRI by inhibiting several processes including, but not limited to, apoptosis, inflammation, oxidative stress and ferroptosis (89). It has been shown that the prophylactic oral administration of Lactobacillus reuteri or its metabolite GABA can mitigate myocardial inflammation mediated by macrophages, thus alleviating MIRI (32). Additionally, Bifidobacterium infantis or its metabolite inosine can exert cardioprotective effects during myocardial I/R by suppressing cardiac inflammation and reducing myocardial cell apoptosis (33). Studies have also revealed that the application of lyophilized S. boulardii and inactivated probiotic Lactobacillus reuteri markedly decreased the myocardial infarct size caused by I/R injury (34). Bulut EC et al. (35). reported that the supplementation of prebiotics and probiotics, alongside a standard diet or a high-fat, high-carbohydrate diet, improved intestinal ecological imbalance, lowered CK-MB and cTnI levels, and mitigated MIRI in hyperglycemic rats.

### Short chain fatty acids treatment

SCFAs, as one of the metabolites of the gut, have been shown to play an important role in the regulation of cardiac function (90). Prior research has indicated that supplementation with acetate, propionate, and butyrate markedly decreased cardiac fibrosis in GPCR (-) mice (36). Studies have also shown that oral administration of butyrate in rats can protect the heart by inhibiting the sympathetic nervous system, thereby reversing the autonomic imbalance caused by myocardial I/R (37). Furthermore, Deng F et al. (38). reported that propionic acid can mitigate CAV-1/ACE2—mediated Ang II-induced MIRI via GPR41, offering a novel therapeutic approach for treating MIRI through the regulation of gut microbiota. The oral administration of engineered probiotics, capable of continuous secretion of short-chain fatty acids, has been demonstrated to be an effective preventative measure against MIRI (91). The role of fatty acids in modulating gut microbiota offers a novel direction for the treatment of MIRI.

### Traditional Chinese medicine treatment

The protective role of TCM in cardiovascular disease has been extensively investigated. Chinese patent medicine, Chinese herb medicine, CM monomer, acupuncture, moxibustion and other treatment methods have shown obvious therapeutic advantages. Currently, certain traditional medications and therapies have demonstrated the ability to mitigate MIRI through the regulation of gut microbiota (92). Research has indicated that FMN, a phytoestrogen belonging to the isoflavone family, can effectively modulate gut microbiota, enhance host metabolism, and reduce cardiac inflammation by inhibiting the ROS-TXNIP-NLRP3 pathway, thereby alleviating MIRI in rats (39). Cui Y et al. (40). discovered that Simiao Yongan decoction, a compound of four Chinese herbs, can ameliorate MIRI in rats by regulating gut microbiota and safeguarding the intestinal barrier, thus reducing the translocation of LPS and inflammatory mediators. There were also studies using electroacupuncture (EA) to intervene in rats with MIRI, and the results showed that the EA intervention could ultimately play a cardioprotective role by improving the damage to the intestinal mucosal barrier, reducing the production of intestinal LPS, and inhibiting myocardial inflammation (7).

## Conclusions

The investigation into the mechanisms, preventive measures, and therapeutic strategies for MIRI remains a significant challenge. The gut microbiota plays various roles in a variety of diseases and has been shown to be closely related to MIRI. In-depth study of the role of the gut microbiota in MIRI is anticipated to enhance our comprehension of myocardial I/R, identify novel prevention and therapeutic targets, and facilitate the development of innovative therapeutic agents. Dysbiosis of the gut microbiota can contribute to the pathogenesis of MIRI and influence its prognosis through mechanisms such as compromising intestinal barrier integrity, stimulating inflammatory, inducing oxidative stress, impairing mitochondrial function, and metabolizing harmful substances. Maintaining normal intestinal barrier function and gut microbiota ecology is one of the important factors to avoid MIRI. High-fiber diet, probiotic consumption, SCFAs supplementation and TCM have demonstrated potential in alleviating MIRI by modulating gut microbial functions, and they have potential preventive and therapeutic effects. however, these preventive and therapeutic strategies cannot be applied to clinical practice because most of the current studies are limited to animal studies and single factors. Consequently, in-depth animal studies, large sample sequencing analyses, and multicenter clinical trials on gut microbiota and MIRI have become increasingly important.

## References

[1] Algoet M Janssens S Himmelreich U Gsell W Pusovnik M Van den Eynde J Myocardial ischemia-reperfusion injury and the influence of inflammation. Trends Cardiovasc Med. (2023) 33(6):357–66. 10.1016/j.tcm.2022.02.005 35181472

[2] Heusch G. Myocardial ischemia/reperfusion: translational pathophysiology of ischemic heart disease. Med. (2024) 5(1):10–31. 10.1016/j.medj.2023.12.007 38218174

[3] Sagris M Apostolos A Theofilis P Ktenopoulos N Katsaros O Tsalamandris S Myocardial ischemia-reperfusion injury: unraveling pathophysiology, clinical manifestations, and emerging prevention strategies. Biomedicines. (2024) 12(4):802. 10.3390/biomedicines12040802 38672157 PMC11048318

[4] Xiang Q Yi X Zhu XH Wei X Jiang DS. Regulated cell death in myocardial ischemia-reperfusion injury. Trends Endocrinol Metab. (2024) 35(3):219–34. 10.1016/j.tem.2023.10.010 37981501

[5] Zhang S Yan F Luan F Chai Y Li N Wang Y-W The pathological mechanisms and potential therapeutic drugs for myocardial ischemia reperfusion injury. Phytomedicine. (2024) 129:155649. 10.1016/j.phymed.2024.155649 38653154

[6] Wang L Wang Y Xu H Li W. Effect of dapagliflozin on ferroptosis through the gut microbiota metabolite TMAO during myocardial ischemia-reperfusion injury in diabetes mellitus rats. Sci Rep. (2024) 14(1):13851. 10.1038/s41598-024-64909-5 38879701 PMC11180094

[7] Bai H Gu RJ Chen LY Qian Y Yu ML Xu SL Electroacupuncture interventions alleviates myocardial ischemia reperfusion injury through regulating gut microbiota in rats. Microvasc Res. (2021) 138:104235. 10.1016/j.mvr.2021.104235 34453991

[8] Braszczyńska-Sochacka J Sochacki J Mik M. The Gut’s secret code: bowel Microbiota as a biomarker for adaptation. Nutrients. (2025) 17(13):2117. 10.3390/nu17132117 40647222 PMC12251568

[9] Yan L Zhang S Lu X Li Z. Gut Microbiota and bipolar disorder: advances in translational applications. Curr Neuropharmacol. (2025). 10.2174/011570159X379789250626044050 PMC13270089 40685737

[10] Xie L Lin W. The role of gut microbiota dysbiosis in the inflammatory pathogenesis of diabetic retinopathy. Front Immunol. (2025) 16:1604315. 10.3389/fimmu.2025.1604315 40692792 PMC12277144

[11] Qiu P Ishimoto T Fu L Zhang J Zhang Z Liu Y. The gut microbiota in inflammatory bowel disease. Front Cell Infect Microbiol. (2022) 12:733992. 10.3389/fcimb.2022.733992 35273921 PMC8902753

[12] Shen J Fang L Wu Y Deng N Peng X Li D Intestinal microbiota dysbiosis disrupts the mucosal barrier, triggering inflammatory responses in gut-kidney interaction and exacerbating diarrhea. J Inflamm Res. (2025) 18:9379–99. 10.2147/JIR.S529493 40687146 PMC12276751

[13] Ronen D Rokach Y Abedat S Qadan A Daana S Amir O Human gut microbiota in cardiovascular disease. Compr Physiol. (2024) 14(3):5449–90. 10.1002/j.2040-4603.2024.tb00298.x 39109979

[14] Tong Y Guo S Li T Yang K Gao W Peng F Gut microbiota and renal fibrosis. Life Sci. (2024) 357:123072. 10.1016/j.lfs.2024.123072 39307181

[15] Schoeler M Caesar R. Dietary lipids, gut microbiota and lipid metabolism. Rev Endocr Metab Disord. (2019) 20(4):461–72. 10.1007/s11154-019-09512-0 31707624 PMC6938793

[16] Zhou Q Chen T Wang X Xu Z Song Y Liu S Role of gut microbiota in neuroinflammation: a focus on perioperative neurocognitive disorders. Front Cell Infect Microbiol. (2025) 15:1582909. 10.3389/fcimb.2025.1582909 40692688 PMC12277274

[17] Xiao K Sun Y Song J Li L Mao W Jiang C. Gut microbiota involved in myocardial dysfunction induced by sepsis. Microb Pathog. (2023) 175:105984. 10.1016/j.micpath.2023.105984 36638851

[18] Weiss GA Hennet T. Mechanisms and consequences of intestinal dysbiosis. Cell Mol Life Sci. (2017) 74(16):2959–77. 10.1007/s00018-017-2509-x 28352996 PMC11107543

[19] Zmora N Suez J Elinav E. You are what you eat: diet, health and the gut microbiota. Nat Rev Gastroenterol Hepatol. (2019) 16(1):35–56. 10.1038/s41575-018-0061-2 30262901

[20] Yang T Maki KA Marques FZ Cai J Joe B Pepine CJ Hypertension and the gut microbiome: a science advisory from the American Heart Association. Hypertension. (2025) 82(9):e160–70. 10.1161/HYP.0000000000000247 40671646

[21] Xu H Yang F Bao Z. Gut microbiota and myocardial fibrosis. Eur J Pharmacol. (2023) 940:175355. 10.1016/j.ejphar.2022.175355 36309048

[22] Forkosh E Ilan Y. The heart-gut axis: new target for atherosclerosis and congestive heart failure therapy. Open Heart. (2019) 6(1):e000993. 10.1136/openhrt-2018-000993 31168383 PMC6519415

[23] Li YL Chen BY Feng ZH Zhou LJ Liu T Lin WZ Roles of oral and gut microbiota in acute myocardial infarction. J Adv Res. (2024) 74:319–32. 10.1016/j.jare.2024.10.009 39447641 PMC12302727

[24] Cheraghi M Nazari A Souri F. Gut microbiota and cardiac arrhythmogenesis: unveiling the gut-heart axis. Pathol Res Pract. ( 2025) 273:156125. 10.1016/j.prp.2025.156125 40675025

[25] Gantenbein KV Kanaka-Gantenbein C. Mediterranean diet as an antioxidant: the impact on metabolic health and overall wellbeing. Nutrients. (2021) 13(6):1951. 10.3390/nu13061951 34204057 PMC8227318

[26] Marques FZ Nelson E Chu PY Horlock D Fiedler A Ziemann M High-fiber diet and acetate supplementation change the gut Microbiota and prevent the development of hypertension and heart failure in hypertensive mice. Circulation. (2017) 135(10):964–77. 10.1161/CIRCULATIONAHA.116.024545 27927713

[27] Kaye DM Shihata WA Jama HA Tsyganov K Ziemann M Kiriazis H Deficiency of prebiotic fiber and insufficient signaling through gut metabolite-sensing receptors leads to cardiovascular disease. Circulation. (2020) 141(17):1393–403. 10.1161/CIRCULATIONAHA.119.043081 32093510

[28] Trinei M Carpi A Menabo R Storto M Fornari M Marinelli A Dietary intake of cyanidin-3-glucoside induces a long-lasting cardioprotection from ischemia/reperfusion injury by altering the microbiota. J Nutr Biochem. (2022) 101:108921. 10.1016/j.jnutbio.2021.108921 34864150

[29] Xu X Lu WJ Shi JY Su Y-l Liu Y-c Wang L The gut microbial metabolite phenylacetylglycine protects against cardiac injury caused by ischemia/reperfusion through activating β2AR. Arch Biochem Biophys. (2021) 697:108720. 10.1016/j.abb.2020.108720 33307065

[30] Zheng D Liu Z Zhou Y Hou N Yan W Qin Y Urolithin B, a gut microbiota metabolite, protects against myocardial ischemia/reperfusion injury via p62/Keap1/Nrf2 signaling pathway. Pharmacol Res. (2020) 153:104655. 10.1016/j.phrs.2020.104655 31996327

[31] Oniszczuk A Oniszczuk T Gancarz M Szymańska J. Role of gut microbiota, probiotics and prebiotics in the cardiovascular diseases. Molecules. (2021) 26(4):1172. 10.3390/molecules26041172 33671813 PMC7926819

[32] Wang J Zhang H Yuan H Chen S Yu Y Zhang X Prophylactic supplementation with lactobacillus reuteri or its metabolite GABA protects against acute ischemic cardiac injury. Adv Sci (Weinh). (2024) 11(18):e2307233. 10.1002/advs.202307233 38487926 PMC11095141

[33] Zhang H Wang J Shen J Chen S Yuan H Zhang X Prophylactic supplementation with Bifidobacterium infantis or its metabolite inosine attenuates cardiac ischemia/reperfusion injury. Imeta. (2024) 3(4):e220. 10.1002/imt2.220 39135700 PMC11316933

[34] Borshchev YY Sinitsa AV Zakharchenko MM Borshchev VY Burovenko IY Galagudza MM. Effect of antiobiotic-induced disbiosis and its correction with probiotics on myocardial tolerance to ischemia-reperfusion injury in SPF rats. Bull Exp Biol Med. (2019) 166(4):440–3. 10.1007/s10517-019-04368-5 30788733

[35] Bulut EC Erol Kutucu D Üstünova S Ağırbaşlı M Dedeakayoğulları H Tarhan Ç Synbiotic supplementation ameliorates anxiety and myocardial ischaemia-reperfusion injury in hyperglycaemic rats by modulating gut microbiota. Exp Physiol. (2024) 109(11):1882–95. 10.1113/EP092052 39264256 PMC11522816

[36] Liu Q Cheng L Wang M Shen L Zhang C Mu J Dietary sodium acetate and sodium butyrate improve high-carbohydrate diet utilization by regulating gut microbiota, liver lipid metabolism, oxidative stress, and inflammation in largemouth bass (Micropterus salmoides). J Anim Sci Biotechnol. (2024) 15(1):50. 10.1186/s40104-024-01009-4 38566217 PMC10988814

[37] Yu Z Han J Chen H Wang Y Zhou L Wang M Oral supplementation with butyrate improves myocardial ischemia/reperfusion injury via a gut-brain neural circuit. Front Cardiovasc Med. (2021) 8:718674. 10.3389/fcvm.2021.718674 34631821 PMC8495014

[38] Deng F Zhang LQ Wu H Chen Y Yu W-Q Han R-H Propionate alleviates myocardial ischemia-reperfusion injury aggravated by angiotensin II dependent on caveolin-1/ACE2 axis through GPR41. Int J Biol Sci. (2022) 18(2):858–72. 10.7150/ijbs.67724 35002530 PMC8741842

[39] Zhang Y Deng J Chen T Liu S Tang Y Zhao JR Formononetin alleviates no reflow after myocardial ischemia-reperfusion via modulation of gut microbiota to inhibit inflammation. Life Sci. (2024) 358:123110. 10.1016/j.lfs.2024.123110 39374772

[40] Cui Y Zhang F Xu W Li Z Zou J Gao P Effects of Si-Miao-Yong-An decoction on myocardial I/R rats by regulating gut microbiota to inhibit LPS-induced TLR4/NF-κB signaling pathway. BMC Complement Med Ther. (2023) 23(1):180. 10.1186/s12906-023-04013-9 37268931 PMC10236840

[41] Cai W Liu L Shi X Yanan L Jin W Xuan F Alox15/15-HpETE aggravates myocardial ischemia-reperfusion injury by promoting cardiomyocyte ferroptosis. Circulation. (2023) 147(19):1444–60. 10.1161/CIRCULATIONAHA.122.060257 36987924

[42] Liu S Bi Y Han T Li Yiran E Qihang W Natalie WN The E3 ubiquitin ligase MARCH2 protects against myocardial ischemia-reperfusion injury through inhibiting pyroptosis via negative regulation of PGAM5/MAVS/NLRP3 axis. Cell Discov. (2024) 10(1):24. 10.1038/s41421-023-00622-3 38409220 PMC10897310

[43] Gao X Ma C Liang S Chen M He Y Lei W. PANoptosis: novel insight into regulated cell death and its potential role in cardiovascular diseases (review). Int J Mol Med. (2024) 54(3):74. 10.3892/ijmm.2024.5398 38963054 PMC11254103

[44] Zhang T Han Y Wang Y Wang X Zhao M Cheng Z The interaction between ferroptosis and myocardial ischemia-reperfusion injury: molecular mechanisms and potential therapeutic targets. Eur J Med Res. (2025) 30(1):643. 10.1186/s40001-025-02851-6 40685346 PMC12278529

[45] Oerlemans MI Liu J Arslan F Ouden K Middelaar BJ Doevendans PA Inhibition of RIP1-dependent necrosis prevents adverse cardiac remodeling after myocardial ischemia-reperfusion *in vivo*. Basic Res Cardiol. (2012) 107(4):270. 10.1007/s00395-012-0270-8 22553001

[46] Wu Q Xu R Zhang K Sun R Yang M Li K Characterization of early myocardial inflammation in ischemia-reperfusion injury. Front Immunol. (2023) 13:1081719. 10.3389/fimmu.2022.1081719 36814859 PMC9939645

[47] Heusch G. Myocardial ischaemia-reperfusion injury and cardioprotection in perspective. Nat Rev Cardiol. (2020) 17(12):773–89. 10.1038/s41569-020-0403-y 32620851

[48] Fan Q Tao R Zhang H Xie H Lu L Wang T Dectin-1 contributes to myocardial ischemia/reperfusion injury by regulating macrophage polarization and neutrophil infiltration. Circulation. (2019) 139(5):663–78. 10.1161/CIRCULATIONAHA.118.036044 30586706

[49] Yap J Cabrera-Fuentes HA Irei J Hausenloy DJ Boisvert WA. Role of macrophages in cardioprotection. Int J Mol Sci. (2019) 20(10):2474. 10.3390/ijms20102474 31109146 PMC6566352

[50] Du Y Duan C Zhang X Shi S Zhu X Lyu M Modulation of NLRP3 inflammasome: advantages of Chinese herbal medicine in treating myocardial ischemia/reperfusion injury. Am J Chin Med. (2025) 53(3):737–69. 10.1142/S0192415X25500284 40374375

[51] Rozich E Ozkurede U Pakkiriswami S Gemilere R Azarin SM Liu JC. Mitochondrial oxidative stress, calcium and dynamics in cardiac ischaemia-reperfusion injury. J Physiol. (2025). 10.1113/JP287770 40448972 PMC13371218

[52] Xiang M Lu Y Xin L Gao J Shang C Jiang Z Role of oxidative stress in reperfusion following myocardial ischemia and its treatments. Oxid Med Cell Longev. (2021) 2021:6614009. 10.1155/2021/6614009 34055195 PMC8149218

[53] He Y Ren S Liu C Zheng X Zhu C. Targeting mitochondrial quality control for myocardial ischemia-reperfusion injury. Mitochondrion. (2025) 84:102046. 10.1016/j.mito.2025.102046 40419068

[54] Cruz-Gregorio A. Targeting ferroptosis via mitochondria dynamics in myocardial ischemia/reperfusion injury. Discov Med. (2025) 37(196):816–27. 10.24976/Discov.Med.202537196.72 40415357

[55] Yang J Zhai Y Huang C Xiang Z Liu H Wu J RP105 attenuates ischemia/reperfusion-induced oxidative stress in the myocardium via activation of the Lyn/Syk/STAT3 signaling pathway. Inflammation. (2024) 47(4):1371–85. 10.1007/s10753-024-01982-y 38568415

[56] Song Z Suo C Liu Y Jin L Xie X Liu J Comprehensive evaluation of non-coding RNA-mediated autophagy regulation in myocardial ischemia-reperfusion injury. Front Pharmacol. (2025) 16:1581341. 10.3389/fphar.2025.1581341 40351430 PMC12062134

[57] Hajiagha MN Taghizadeh S Asgharzadeh M Dao S Ganbarov K Köse Ş Gut microbiota and human body interactions; its impact on health: a review. Curr Pharm Biotechnol. (2022) 23(1):4–14. 10.2174/1389201022666210104115836 33397232

[58] Rahman MM Islam F Or-Rashid MH Mamun AA Rahaman MS Islam MM The gut microbiota (microbiome) in cardiovascular disease and its therapeutic regulation. Front Cell Infect Microbiol. (2022) 12:903570. 10.3389/fcimb.2022.903570 35795187 PMC9251340

[59] Nowiński A Ufnal M. Gut bacteria-derived molecules as mediators and markers in cardiovascular diseases. The role of the gut-blood barrier. Kardiol Pol. (2018) 76(2):320–7. 10.5603/KP.a2017.0204 29131297

[60] Tang TWH Chen HC Chen CY Yen CYT Lin C-J Prajnamitra RP Loss of gut microbiota alters immune system composition and cripples postinfarction cardiac repair. Circulation. (2019) 139(5):647–59. 10.1161/CIRCULATIONAHA.118.035235 30586712

[61] Liu Q Zhu Y Li G Guo T Jin M Xi D Irisin ameliorates myocardial ischemia-reperfusion injury by modulating gut microbiota and intestinal permeability in rats. PLoS One. (2023) 18(9):e0291022. 10.1371/journal.pone.0291022 37656700 PMC10473488

[62] Cheng G Zhang J Jia S Feng P Chang F Yan L Cardioprotective effect of gossypin against myocardial ischemic/reperfusion in rats via alteration of oxidative stress, inflammation and gut Microbiota. J Inflamm Res. (2022) 15:1637–51. 10.2147/JIR.S348883 35282267 PMC8906873

[63] Foerster EG Mukherjee T Cabral-Fernandes L Rocha JDB Girardin SE Philpott DJ. How autophagy controls the intestinal epithelial barrier. Autophagy. (2022) 18(1):86–103. 10.1080/15548627.2021.1909406 33906557 PMC8865220

[64] Ullah H Arbab S Tian Y Liu C-q Chen Y Qijie L The gut microbiota-brain axis in neurological disorder. Front Neurosci. (2023) 17:1225875. 10.3389/fnins.2023.1225875 37600019 PMC10436500

[65] Kalyan M Tousif AH Sonali S Ray B Gorantla VR Rungratanawanich W Role of endogenous lipopolysaccharides in neurological disorders. Cells. (2022) 11(24):4038. 10.3390/cells11244038 36552802 PMC9777235

[66] Zhao J Zhang Q Cheng W Dai Q Wei Z Guo M Heart-gut microbiota communication determines the severity of cardiac injury after myocardial ischaemia/reperfusion. Cardiovasc Res. (2023) 119(6):1390–402. 10.1093/cvr/cvad023 36715640 PMC10262181

[67] Di Vincenzo F Del Gaudio A Petito V Lopetuso LR Scaldaferri F. Gut microbiota, intestinal permeability, and systemic inflammation: a narrative review. Intern Emerg Med. (2024) 19(2):275–93. 10.1007/s11739-023-03374-w 37505311 PMC10954893

[68] Chen C Zhang H Xie R Wang Y Ma Y. Gut microbiota aggravate cardiac ischemia-reperfusion injury via regulating the formation of neutrophils extracellular traps. Life Sci. (2022) 303:120670. 10.1016/j.lfs.2022.120670 35640777

[69] Wang W Zhu LJ Leng YQ Wang Y-W Shi T Wang W-Z Inflammatory response: a crucial way for gut microbes to regulate cardiovascular diseases. Nutrients. (2023) 15(3):607. 10.3390/nu15030607 36771313 PMC9921390

[70] Rath E Moschetta A Haller D. Mitochondrial function - gatekeeper of intestinal epithelial cell homeostasis. Nat Rev Gastroenterol Hepatol. (2018) 15(8):497–516. 10.1038/s41575-018-0021-x 29844587

[71] Su X Zhou M Li Y Zhang J An N Yang F Protective effects of natural products against myocardial ischemia/reperfusion: mitochondria-targeted therapeutics. Biomed Pharmacother. (2022) 149:112893. 10.1016/j.biopha.2022.112893 35366532

[72] Muttiah B Hanafiah A. Gut microbiota and cardiovascular diseases: unraveling the role of dysbiosis and microbial metabolites. Int J Mol Sci. (2025) 26(9):4264. 10.3390/ijms26094264 40362500 PMC12072866

[73] Praveenraj SS Sonali S Anand N Tousif HA Vichitra C Kalyan M The role of a gut microbial-derived metabolite, Trimethylamine N-Oxide (TMAO), in neurological disorders. Mol Neurobiol. (2022) 59(11):6684–700. 10.1007/s12035-022-02990-5 35986843

[74] Saeedi Saravi SS. Metabolites matter for gut microbiota as a modifiable risk factor in cardiovascular diseases. Nat Rev Cardiol. ( 2025) 22(9):610. 10.1038/s41569-025-01193-4 40681706

[75] Chen ML Yi L Zhang Y Zhou X Ran L Yang J Resveratrol attenuates Trimethylamine-N-Oxide (TMAO)-induced atherosclerosis by regulating TMAO synthesis and bile acid metabolism via remodeling of the gut Microbiota. mBio. (2016) 7(2):e02210–e2215. 10.1128/mBio.02210-15 27048804 PMC4817264

[76] Wang Z Roberts AB Buffa JA Levison B Zhu W Org E Non-lethal inhibition of gut microbial trimethylamine production for the treatment of atherosclerosis. Cell. (2015) 163(7):1585–95. 10.1016/j.cell.2015.11.055 26687352 PMC4871610

[77] Baginski AM Farmer N Baumer Y Wallen GR Powell-Wiley TM. Interleukin-8 (IL-8) as a potential mediator of an association between Trimethylamine N-Oxide (TMAO) and proprotein convertase Subtilisin/Kexin type 9 (PCSK9) among African Americans at risk of cardiovascular disease. Metabolites. (2022) 12(12):1196. 10.3390/metabo12121196 36557234 PMC9785610

[78] Fusco W Lorenzo MB Cintoni M Porcari S Rinninella E Kaitsas F Short-chain fatty-acid-producing Bacteria: key components of the human gut Microbiota. Nutrients. (2023) 15(9):2211. 10.3390/nu15092211 37432351 PMC10180739

[79] González-Correa C Moleón J Miñano S Robles-Vera I Toral M Martín-Morales N Mineralocorticoid receptor blockade improved gut microbiota dysbiosis by reducing gut sympathetic tone in spontaneously hypertensive rats. Biomed Pharmacother. (2023) 158:114149. 10.1016/j.biopha.2022.114149 36566524

[80] Bridgeman S Woo HC Newsholme P Mamotte C. Butyrate lowers cellular cholesterol through HDAC inhibition and impaired SREBP-2 signalling. Int J Mol Sci. (2022) 23(24):15506. 10.3390/ijms232415506 36555149 PMC9779842

[81] Seefeldt JM Homilius C Hansen J Lassen TR Jespersen NR Jensen RV Short-chain fatty acid butyrate is an inotropic agent with vasorelaxant and cardioprotective properties. J Am Heart Assoc. (2024) 13(9):e033744. 10.1161/JAHA.123.033744 38686853 PMC11179878

[82] Wang X Dong Y Huang R Wang F Xie J Liu H The role of short-chain fatty acids in myocardial ischemia-reperfusion injury. Curr Nutr Rep. (2024) 13(4):701–8. 10.1007/s13668-024-00564-6 39110372 PMC11489193

[83] Allam-Ndoul B Castonguay-Paradis S Veilleux A. Gut microbiota and intestinal trans-epithelial permeability. Int J Mol Sci. (2020) 21(17):6402. 10.3390/ijms21176402 32899147 PMC7503654

[84] Becattini S Taur Y Pamer EG. Antibiotic-induced changes in the intestinal microbiota and disease. Trends Mol Med. (2016) 22(6):458–78. 10.1016/j.molmed.2016.04.003 27178527 PMC4885777

[85] Trasande L Blustein J Liu M Corwin E Cox LM Blaser MJ. Infant antibiotic exposures and early-life body mass. Int J Obes. (2013) 37(1):16–23. 10.1038/ijo.2012.132 PMC3798029 22907693

[86] Mu X Feng L Wang Q Li H Zhou H Yi W Decreased gut microbiome-derived indole-3-propionic acid mediates the exacerbation of myocardial ischemia/reperfusion injury following depression via the brain-gut-heart axis. Redox Biol. (2025) 81:103580. 10.1016/j.redox.2025.103580 40058066 PMC11930714

[87] Du H Liu X Shen J Yuan H Zhang H Xi G Flavonifractor plautii or its metabolite desaminotyrosine as prophylactic agents for alleviating myocardial ischemia/reperfusion injury. Adv Sci. (2025) 12(21):e2417827. 10.1002/advs.202417827 PMC12140293 40089859

[88] Perler BK Friedman ES Wu GD. The role of the gut microbiota in the relationship between diet and human health. Annu Rev Physiol. (2023) 85:449–68. 10.1146/annurev-physiol-031522-092054 36375468

[89] Liang Y Zhao L Zhang X Liu S Lu P Wang J Lactobacillus ameliorates myocardial ischemia reperfusion injury by attenuating apoptosis, inflammation, oxidative stress, and ferroptosis. BMC Med. (2025) 23(1):377. 10.1186/s12916-025-04203-x 40598393 PMC12218948

[90] Hu T Wu Q Yao Q Jiang K Yu J Tang Q. Short-chain fatty acid metabolism and multiple effects on cardiovascular diseases. Ageing Res Rev. (2022) 81:101706. 10.1016/j.arr.2022.101706 35932976

[91] Pham QH Bui TVA Sim WS Lim KH Law COK Tan W Daily oral administration of probiotics engineered to constantly secrete short-chain fatty acids effectively prevents myocardial injury from subsequent ischaemic heart disease. Cardiovasc Res. (2024) 120(14):1737–51. 10.1093/cvr/cvae128 38850165 PMC11587561

[92] Zhang S Zhao Y Ma QZ Li N Chen ZL Fu RJ Role of Chinese medicine in addressing myocardial ischemia reperfusion injury: a comprehensive review. Chin J Integr Med. (2025). 10.1007/s11655-025-4011-x 40447927

[93] Videja M Vilskersts R Sevostjanovs E Liepinsh E Dambrova M. Data on cardiac and vascular functionality in ex vivo and in vivo models following acute administration of trimethylamine N-oxide. Data Brief. (2023) 46:108890. 10.1016/j.dib.2023.108890 36687149 PMC9851877

